# Does the EQ-5D usual activities dimension measure what it intends to measure? The relative importance of work, study, housework, family or leisure activities

**DOI:** 10.1007/s11136-020-02501-w

**Published:** 2020-04-23

**Authors:** Thor Gamst-Klaussen, Admassu N. Lamu

**Affiliations:** 1 Department of Psychology, UiT The Arctic University of Norway, 9037 Tromsø, Norway; 2 Department of Global Public Health and Primary Care, University of Bergen, 5020 Bergen, Norway

**Keywords:** EQ-5D, Usual activities, HRQoL, Shapley value, Relative importance

## Abstract

**Background:**

The EQ-5D is the most widely used generic preference-based health-related quality of life measure. It comprises five dimensions: mobility, self-care, usual activities, pain/discomfort and anxiety/depression. The usual activities dimension asks respondents to evaluate the severity of problems in their usual activities, such as work, study, housework, family or leisure activities. The primary aim of this study is to investigate whether the EQ-5D (five-level) usual activities dimension captures those activities that it intends to capture. We further assess the relative importance of each of these activities for the usual activities dimension.

**Methods:**

Data include 7933 respondents from six countries: Australia, Canada, Germany, Norway, the UK, and the US. Logistic regression and ordinary least square regression models investigate the relationship between the usual activities dimension and its main predictors (work/study, housework, family, and leisure activities). A Shapley value decomposition method was applied to measure the relative importance of each predictor.

**Results:**

Work/study, housework, family, and leisure activities were all significant (*p* < 0.001) determinants of usual activities dimension. The respective marginal contribution (in %) of housework, leisure, work/study and family to UA dimension (as a share of goodness-of-fit) is 28.0, 26.2, 20.8 and 14.7 in the logistic regression model. This finding is consistent when linear regression is used as an alternative model.

**Conclusions:**

The usual activities dimension in EQ-5D reflects the specific activities described to respondents. Therefore, the usual activities dimension measures what it really intends to measure.

**Electronic supplementary material:**

The online version of this article (10.1007/s11136-020-02501-w) contains supplementary material, which is available to authorized users.

## Introduction

Nowadays, the Euroqol 5-dimensional questionnaire, usually termed as EQ-5D, is the most widely used generic preference-based health-related quality of life measure [1–3]. Although EQ-5D has been developed to produce a preference-based index for economic evaluations, it has been widely used across different settings, such as individual health status for use in clinical settings, observational studies, population health surveys [3]. The EQ-5D descriptive system initially comprised of six dimensions that included two dimensions asking respondents to report problems with: (i) ‘Main activity’ i.e., able to perform main activity (e.g., *work, study, housework*); and (ii) ‘Social relationships’ i.e., able to pursue *family* and *leisure* activities, as well as the other four dimensions of the current version of EQ-5D (mobility, self-care, pain/discomfort, and anxiety/depression) [4]. However, the initial experimentation with this six-dimensional version resulted in the merging of ‘Social relationships’ with ‘Main activity’ dimension that became the current ‘UA’ dimension, which leads to the present five-dimensional questionnaire. This change was accompanied by an explicit description of the UA dimension as work, study, and housework alongside family and leisure activities [4]. There was a broad agreement amongst the EuroQol group that the UA dimension would cover the described daily activities, where ‘usual’ was perceived as referring to some frequency or regularity of performance of the activities [5].

Nevertheless, practitioners have raised concerns about the UA dimension due to either respondents’ perceived ambiguity when interpreting the UA dimension or respondents’ counter-intuitive responses [5]. Selai [5] summarized possible sources of ambiguity, including *semantics* (usual/unusual may refer to regularity/frequency or to normal versus abnormal); *vague quantifiers* (relative frequency conveys different meaning depending on race, education, and age); and the *double* or *multi-barrelled* nature of the question (e.g., how to answer if you cannot work but do not have any problems doing leisure activities like reading or playing chess). Furthermore, in adaption to long-lasting chronic illness, one’s usual activities that were initially affected by the disease become less regular and replaced by new daily or frequent activities through activity adjustment [6]. This might make the UA dimension especially prone to adaption that would potentially affect the responses of individuals with chronic conditions.

In general, the UA dimension is thought to measure both activities and social participation [7, 8]. However, there is mixed evidence from the literature. For example, research among stroke patients suggested that the UA dimension was measuring social functioning [5, 9], while a study among patients with diabetes indicated that the UA dimension measure limitation with work but not social activities [10]. Lin et al. [11] also showed that UA dimension correlated strongly with subscales on the Patient-Reported Outcome Measurement Information System (PROMIS), such as physical function, fatigue, and satisfaction with social roles. It has also been suggested that interpersonal relationships might be important as a bolt-on dimension [12, 13].

Moreover, evidence showed that the UA dimension is associated with both physical and mental aspects of health, but more strongly with the former. For instance, using data comprising seven disease groups and a non-diagnosed healthy group, Gamst-Klaussen et al. revealed stronger correlations between UA dimension and the other EQ-5D dimensions that tap physical health (i.e., mobility, self-care and pain/discomfort) than with anxiety/depression [14]. A similar result was found in a study among individuals with COPD [11]. A study that described the most commonly reported EQ-5D health states using SF-36 in the general Swedish population showed that moderate problems on EQ-5D UA dimension most strongly affected the SF-36 role limitations scales due to physical health problems [15]. A comparison of SF-12 and EQ-5D administered in the US general population also demonstrated that the UA dimension had a stronger relationship with the SF-12 physical health component than the SF-12 mental health component [16].

The UA dimension generally reflects a wider concept that may include several activities. The important question is whether the UA dimension captures activities that are described in the EQ-5D questionnaire. To the best of our knowledge, no study has investigated this research question. Thus, the primary aims of the current study are twofold: (i) to investigate whether the described activities (e.g. *work, study, housework, family or leisure activities*) actually determine the EQ-5D UA dimension; and (ii) to assess the relative importance of each of these activities for the UA dimension. We also check the consistency of our findings using samples from seven disease groups and the healthy group.

## Method

### Data

Data were obtained from the multi-instrument comparison (MIC) study, which is based on a 2011/2012 online survey from six countries (Australia, Canada, Germany, Norway, UK, US) and administered by a global panel company, CINT Pty Ltd. The data include a sample of 7933 respondents comprising a ‘healthy group’ (*N* = 1760) and seven major disease groups (*N* = 6173). Respondents were initially asked to indicate if they had a chronic disease and to rate their overall health on a visual analogue scale (VAS). Quotas on age, gender, and education were used to obtain a demographically representative sample of a *healthy group*, defined by the absence of chronic disease and a VAS score of at least 70 on overall health. Quotas were also applied to obtain a target number of respondents in each disease group (arthritis, asthma, cancer, depression, diabetes, hearing loss, heart diseases). See Table 1 for the description of data.

**Table 1:** Number of respondents by country and disease group

Diseases	Australia	UK	USA	Canada	Norway	Germany	Total	%
Asthma	141	150	150	138	129	147	855	10.7
Cancer	154	137	148	138	80	115	772	10.0
Depression	146	158	168	145	140	160	917	12.0
Diabetes	168	161	168	144	143	140	924	12.0
Hearing loss	155	126	156	144	113	136	830	10.5
Arthritis	163	159	179	139	130	159	929	12.0
Heart disease	149	167	170	154	151	152	943	12.0
Healthy group	265	298	321	328	288	260	1760	22.0
Total	1341	1356	1460	1330	1174	1269	7933	100.0

The MIC survey includes six generic preference-based measures and a generic health status measure, as well as several disease-specific and well-being measures. To avoid priming the respondents’ feelings with questions about their health, which could potentially lead to biased responses, the subjective well-being questions were first administered. Next, EQ-5D and other generic measures were administered in a randomized order to avoid order effects. Finally, the respondents in each disease-group completed a disease-specific measure. Responses were subject to several stringent edit procedures based upon a comparison of duplicated or similar questions as well as a minimum completion time to ensure the quality of data. The detailed edit procedures and its administrations are available elsewhere [17].

### Measures of variables

#### Outcome variable

The EQ-5D has five dimensions: mobility, self-care, usual activities, pain/discomfort, and anxiety/depression. Each dimension has three severity levels in the original version and five severity levels in the revised version. Here, we considered the latter version, EQ-5D five-levels. The UA dimension of EQ-5D is the outcome or dependent variable and has five response levels: no problems, slight problems, moderate problems, severe problems, extreme problems. A closer examination reveals that all except the first level have one common feature: having problems with usual activities (though with different degrees of severity). Thus, the UA dimension can logically be dichotomized into full health and impaired health. That is, level-1—“I have no problems doing my usual activities”—represents full health, while the rest (level-2 to level-5) represent impaired health.

#### Predictor variables

Relevant items were identified from Medical Outcomes Study 36-item Short-Form (SF-36) questionnaire and the Assessment of Quality of Life (AQoL) questionnaire that would likely measure the main predictors: work/study, housework, family, and leisure activities.

##### Work/study activities

Seven items from SF-36 were selected, which would likely measure problems related to *work/study* activities due to one's health. Four items were related to physical health, and the remaining three items ask about emotional problems. The question related to physical health is described as: *During the past 4 weeks, have you had any of the following problems with your work or other regular daily activities as a result of your PHYSICAL health?*

(i) Cut down the amount of time you spent on work or other activities;
(ii) Accomplished less than you would like
(iii) Were limited in the kind of work or other activities;
(iv) had difficulty performing work or other activities (for example, it took extra effort).

The emotional question is stated as: *During the past 4 weeks, have you had any of the following problems with your work or other regular daily activities as a result of your EMOTIONAL problems (such as feeling depressed or anxious)*?

(i) Cut down the amount of time you spent on work or other activities;
(ii) Accomplished less than you would like;
(iii) Did not do work or other activities as carefully as usual.

Responses for each of the seven items from physical and emotional health is given on a five-point Likert scale (reverse coded): None of the time; A little of the time; Some of the time; Most of the time; All of the time. The total summary score of the seven items was used as a measure of work/study activities and treated as a continuous variable with higher values indicating greater problems in performing work/study activities. To check the consistency of our results, one item from EORTC Quality of Life Questionnaire (QLQ C-30) with a similar measure of *work/study* activity was applied using a cancer sample (*N* = 772). It is described as: *were you limited doing either your work or other daily activities?* (Not at all; A little; Quite a bit; Very much)*.*

##### Housework activities

One item from AQoL with five response levels was selected to measure *housework* activities, and defined as: *How much help do you need with jobs around the house (e.g., preparing food, cleaning the house or gardening)*? The responses include: I can do all these tasks very quickly and efficiently without any help; I can do these tasks relatively easily without help; I can do these tasks only very slowly without help; I cannot do most of these tasks unless I have help; I can do none of these tasks by myself.

Because only a few responses were observed on the last scale (0.5%), the last two levels were combined. Thus, housework is treated as a categorical variable with four levels.

##### Family activities

One item from AQoL was selected to measure *family* activities. The item has four response levels, and described as follows *Thinking about your health and your relationship with your family;* my role in the family is unaffected by my health; there are some parts of my family role I cannot carry out; there are many parts of my family role I cannot carry out; I cannot carry out any part of my family role. The two most severe levels were merged due to few respondents on the last level (1.3%).

##### Leisure activities

Two items from SF-36 (each with three response levels) were selected as a measure of *leisure* activities*.* That is, respondents are asked to consider if their health limits them in the following activities, and if so how much.

(i) *Vigorous activities, such as running, lifting heavy objects, participating in strenuous sports* (Yes, limited a lot; Yes, limited a little; Not limited at all).
(ii) *Moderate activities, such as moving a table, pushing a vacuum cleaner, bowling, or playing golf* (Yes, limited a lot; Yes, limited a little; Not limited at all).

The total score was calculated by summing the reverse-coded responses across the two items. Eventually, this variable is grouped into five categories ranging from 1 (not limited at all) to 5 (limited a lot) in the full sample. Because only a few responses were observed on the first level, the first two levels were combined in the sample of the healthy group, asthma, and hearing problems.

The descriptions of these two items go beyond leisure activities because they illustrate physical and housework activities as well. Thus, to check the consistency of our results, a similar analysis was conducted using a sample of cancer patients (*N* = 772) who reported their HRQoL using QLQ C-30 that includes one item with a better measure of *leisure* activity. The item asks specifically about hobbies and other leisure activities and has four response levels. It is described as: *were you limited pursuing your hobbies or other leisure activities?* (Not at all; A little; Quite a bit; Very much)*.*

##### Control variables

In addition to the described main predictors, we considered the effect of socio-demographic characteristics. Age is included as a continuous variable to control any variation in UA due to age differences. Gender (0 = male; 1 = female) is used to control sex differences. Marital status may also be an important determinant of UA (0 = no partner/spouse, 1 = partner/spouse). Education level is accounted for by dummies (0 = high school; 1 = diploma or certificate; 2 = university). Employment status is dichotomized (*unemployed* vs. *all others*) to reflect the evidence that being unemployed has a particularly adverse effect on usual activities dimension. Furthermore, disease variables are included since they may signal the effect of health variations on UA and country variables to capture country-specific heterogeneity. Description of variables is summarized in Table 2.

**Table 2:** Description of variables

Variable	Mean/*n*	SD/%
Age (years)	51.46	15.41
Gender, *n*(%)		
Female	4140	52.19
Male	3793	47.81
Marital status, *n*(%)		
Live with partner/spouse	5085	64.1
Do not live with partner/spouse	2848	35.9
Education level, *n*(%)		
High school	2482	31.29
Diploma/certificate	3208	40.44
University	2243	28.27
Employment status, *n*(%)		
Unemployed	607	7.65
Employed/other	7323	92.35
Usual activities, *n*(%)		
Have no problems	5163	65.08
Have problems	2770	34.92
Work/study	13.40	7.60
Housework, *n*(%)		
Can do all tasks very quickly	*3912*	49.31
Can do relatively easily	2475	31.2
Can do very slowly	1079	13.6
Cannot do most unless help/none	467	5.89
Family, *n*(%)		
My family role is unaffected	5288	66.66
I cannot carry out some parts	1957	24.67
I cannot carry out many parts/any parts	688	8.67
Leisure, *n*(%)		
Not limited	2095	26.41
Slightly limited	2148	27.08
Somewhat limited	1279	16.12
Moderately limited	1516	19.11
Limited a lot	894	11.27

### Statistical analyses

Respondents’ characteristics were described as mean (standard deviation, SD) unless otherwise indicated. The Spearman rank-order correlation analysis was used to test the association between UA dimension and the main predictors. Spearman's correlation coefficient (*ρ*) measures the strength and direction of the monotonic association between two variables. It is a nonparametric rank statistic often used to evaluate relationships involving ordinal variables without making any assumptions about the frequency distribution of the variables [18].

Logistic regression model (LRM), which is the most commonly used for a binary outcome, was applied to investigate the effect of main predictors (work/study, housework, family, and leisure activities) on UA dimension of EQ-5D. Standard ordinary least square (OLS) regression results were also presented to facilitate comparison with the LRM, which enables us to test the stability of our results. In the OLS, the UA dimension with five severity levels was used as a continuous variable. All explanatory variables were tested for multi-collinearity and were found to be satisfactory as their maximum Variance Inflation Factors (VIFs) were below 3.0, which is much less than the generally accepted maximum threshold value of 10 [19].

In both models, we first tested the significance of the main predictors in explaining the UA dimension. Then we investigated the relative contribution of each of the main predictors for UA dimension using the variance decomposition approach. The *variance decomposition* method, referred to as the Shapley value (SV) regression, was employed to detect the relative importance of each predictor (work/study, housework, family, and leisure activities) for UA dimension. In health research with inherently imprecise measures of complex concepts such as quality of life or self-reported health, a correlation among predictors is often the norm [20]. Therefore, the SV regression [21] is a reliable and stable method for the estimation of predictor importance even in the presence of high multi-collinearity. The SV measures the marginal contribution to the explained variance (*R*^2^) from adding a given independent variable to the model, weighted by the number of permutations represented by this sub-model [22]. A detailed description of SV is found elsewhere [20, 22].

Model estimates from a logistic regression are based on maximum likelihood estimates retrieved through an iterative process. Unlike the OLS *R*^2^, several pseudo *R*^2^ have been developed to evaluate the goodness-of-fit of logistic models. Because the interpretation of pseudo *R*^2^ is not identical to the *R*^2^ in OLS regression (the proportion of variance explained by the predictors), we suggest caution in interpreting it. Nevertheless, in this study, we used McFadden’s pseudo *R*^2^ [23], which is the most commonly used and more straightforward in the sense that it reflects both the criterion being minimized in logistic regression estimation and the variance-accounted for by the LRM [24]. Although the Pseudo-*R*^2^ from LRM is not directly comparable with the OLS *R*^2^, the SV approach can decompose the total explained variation from both LRM and OLS.

## Results

Summary statistics of the variables were presented in Tables 1 and 2. Almost 65% of individuals reported no problems in doing their UA activities, while those with no problems in family, housework, leisure, and work/study activities was 66.7, 49.3, 30.7, and 26.4%, respectively. The mean age of respondents was 51.46 (SD = 15.41) years, and ranged 18–93 years. Data were fairly gender-balanced (52% female). Most respondents had a diploma or university degree (68%), the majority lived with a partner or spouse (64%), and the distributions of chronic diseases were fairly balanced in the sample.

The Spearman correlation coefficient between the UA dimension and the main predictors were quite high: with work/study (rho, *ρ* = 0.63), housework (*ρ* = 0.66), family (*ρ* = 0.57) and leisure activities (*ρ* = 0.63). The Spearman’s correlation coefficient tends to yield a better correlation estimate than a Pearson product-moment correlation applied to ordinal variables, especially when the distribution of the ordinal variables is skewed.

The regression results were presented in Table 3. All main predictors, including work/study, housework, family, and leisure activities were significant (*p* < 0.001) determinants of UA dimension. For instance, results from the LRM revealed that the odds of *having problems* in UA was 12 (= *e*^2.5^) times higher for those who are unable to do the housework tasks than those who are able to do the housework tasks very quickly. Similarly, OLS results showed that being unable to do most or none of the housework tasks without help leads to 0.85 higher problems in doing UA as compared to those who can do housework tasks very quickly. In the OLS, being ‘slightly limited’ to do leisure activities reduced UA by 0.6-percentage point as compared to ‘not limited at all’ but was not statistically significant; however, the LRM significantly picked up the difference. Among control variables, having a chronic condition significantly determined UA. Both LRM and OLS models generally produced similar results in the sense that in both models the regression results showed the same directions. Both models also produced similar ranks with regard to the importance of activities that describe the UA dimension (as detailed in the next paragraph). Results from the cancer sample with a more precise definition of leisure variable demonstrated similar results (Online Appendix Table A1), and so does the proxy variable for work/study (Online Appendix Table A2).

**Table 3:** Regression results for usual activities dimension

Variables	Model 1				Model 2			
	*β*	S.E.	95% CI		*β*	S.E.	95% CI	
			Lower	Upper			Lower	Upper
Work/study	0.093***	0.006	0.08	0.105	0.027***	0.001	0.024	0.029
Housework								
Can do relatively easy	1.011***	0.085	0.845	1.178	0.124***	0.015	0.095	0.152
Can do very slowly	2.186***	0.132	1.927	2.445	0.568***	0.031	0.507	0.630
Cannot do most/none	2.475***	0.246	1.993	2.957	0.853***	0.051	0.753	0.954
Family								
Some parts affected	0.776***	0.083	0.612	0.939	0.133***	0.019	0.095	0.170
Many/cannot cary any	0.934***	0.165	0.612	1.257	0.469***	0.04	0.391	0.548
Leisure								
Slightly limited	0.639***	0.127	0.392	0.887	0.006	0.011	− 0.016	0.028
Somewhat limited	1.314***	0.133	1.053	1.575	0.093***	0.019	0.056	0.130
Moderately limited	2.014***	0.135	1.748	2.279	0.273***	0.024	0.227	0.319
Limited a lot	2.104***	0.185	1.740	2.467	0.588***	0.041	0.507	0.668
Age (in years)	− 0.0005	0.003	− 0.006	0.005	− 0.0001	0.0004	− 0.001	0.001
Gender								
Female	− 0.028	0.076	− 0.177	0.121	− 0.013	0.012	− 0.036	0.011
Marital								
Live with spouse/partner	− 0.084	0.075	− 0.231	0.064	− 0.012	0.012	− 0.036	0.013
Education								
Diploma/certificate	− 0.140*	0.085	− 0.306	0.026	− 0.026*	0.014	− 0.054	0.002
University	− 0.226**	0.095	− 0.412	− 0.039	− 0.037**	0.015	− 0.066	− 0.008
Employment status								
Unemployed	0.097	0.139	− 0.175	0.368	0.017	0.026	− 0.033	0.068
Disease								
Arthritis	1.284***	0.146	0.998	1.571	0.149***	0.022	0.106	0.192
Asthma	0.628***	0.152	0.330	0.925	0.051***	0.018	0.015	0.087
Cancer	0.816***	0.152	0.518	1.114	0.107***	0.022	0.063	0.151
Depression	0.925***	0.154	0.623	1.226	0.088***	0.025	0.039	0.136
Diabetes	0.543***	0.148	0.253	0.833	0.048**	0.019	0.010	0.085
Hearing	0.500***	0.162	0.182	0.819	0.032*	0.018	− 0.003	0.067
Heart	0.738***	0.147	0.450	1.026	0.079***	0.02	0.040	0.119
Country								
Australia	− 0.229*	0.127	− 0.477	0.020	− 0.102***	0.02	− 0.142	− 0.062
Canada	− 0.122	0.129	− 0.376	0.132	− 0.037*	0.02	− 0.076	0.002
Germany	0.300**	0.129	0.048	0.553	0.033	0.021	− 0.008	0.074
Norway	0.178	0.132	− 0.081	0.437	0.003	0.02	− 0.036	0.042
USA	− 0.139	0.126	− 0.387	0.108	− 0.053***	0.02	− 0.092	− 0.014
Constant	− 10.700***	0.583	− 11.843	− 9.557	− 0.141***	0.077	− 0.2923	0.011075

Logistic regression is used in Model 1, and linear regression in Model 2

*β* estimated coefficients, *S.E.* standard error, *CI* confidence interval

***p < 0.01, **p < 0.05, *p < 0.1

**Fig. 1:**
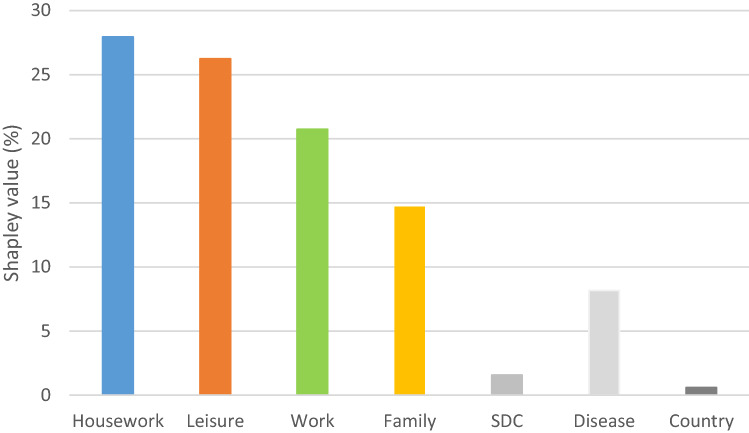
Relative importance of predictors in the full sample (*N* = 7933). Note: *SDC* Socio-demographic characteristics (age, gender, marital, education and unemployment)

Figure 1 and Table 4 summarize the relative importance of each predictor for UA dimension of EQ-5D. The overall goodness-of-fit was over 49 and 63% in LRM and OLS, respectively. Housework, leisure, work/study, and family activities significantly contributed to the total explained variation in the UA dimension, in that order. For instance, the respective marginal contribution of housework, leisure, work/study, and family activities to UA dimension (as a share of goodness-of-fit) was 28.0, 26.2, 20.8, and 14.7% in the LRM. Similar results observed in the OLS regression model: housework contributed the highest (28.7%) followed by leisure (24.1%). Family activities contributed the least (17.9%).

**Table 4:** Relative importance of predictors for EQ-5D usual activities dimension

Predictors	Full sample (*n* = 7933)			
	Model 1		Model 2	
	Est	Percent	Est	Percent
Work/study	0.102	20.75	0.145	22.85
Housework	0.137	27.95	0.182	28.74
Family	0.072	14.71	0.113	17.89
Leisure	0.129	26.24	0.153	24.11
SDC	0.008	1.58	0.007	1.07
Disease	0.040	8.18	0.030	4.73
Country	0.003	0.60	0.004	0.62
Total	0.491	100	0.633	100

Model 1 applied logistic regression, and Model 2 linear regression

*SDC* socio-demographic characteristics (age, gender, marital, education abd unemployment), *Est*. estimated shapley value

Analysis using a sample of cancer patients (*N* = 772) with a more precise measure of leisure activities produced similar results, except that work/study became the second-most important for UA dimension instead of leisure (Online Appendix Table A3, Scenario 1). In the LRM, the relative contributions of housework, work/study, leisure, and family activities to UA dimension of the EQ-5D were 37.8, 23.4, 19.9, and 15.9%, respectively. A similar pattern was observed from the OLS regression model. When we consider an alternative measure of work/study variable from the cancer sample, the LRM produced identical importance rankings; i.e., housework is the most important variable followed by leisure similar to the model with full sample. Family variable ranked last. However, leisure and work/study switched in the OLS model. For detail, see Online Appendix Table A3.

**Fig. 2:**
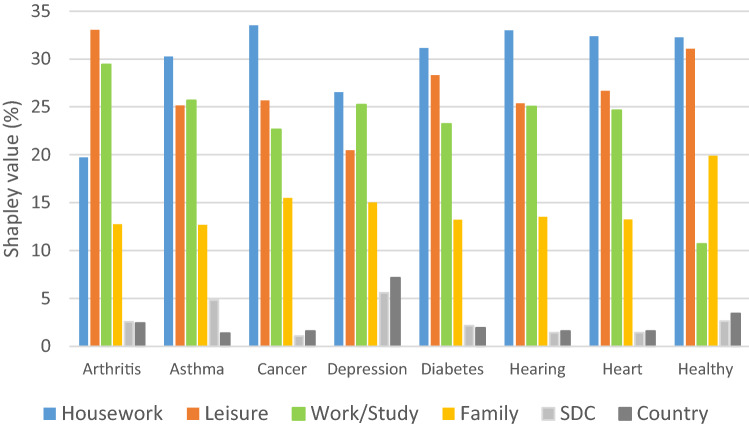
Relative importance of predictors in disease groups and healthy group. Note: *SDC* Socio-demographic characteristics (age, gender, marital, education and unemployment)

Figure 2 and Online Appendix Table A4 summarized the relative importance of the main predictors of UA dimension across the *healthy group* and disease groups. The relative importance rankings were quite similar across different groups. Housework was the most important variable except in the arthritis group, where leisure contributed most. Family activities consistently ranked least except in the healthy group where work/study and family activities switched importance rankings. In addition, the depression group differs in two ways: first, control variables were most strongly associated with the UA dimension; and second, it had the lowest goodness-of-fit (i.e., *R*^2^ = 38%). It is also worth mentioning that the marginal contribution of work/study exceeds that of leisure in the depression group alone.

## Discussion

This study investigated whether the EQ-5D usual activities dimension reflects measures of variables related to work/study, housework, family, and leisure activities as described in the questionnaire. The findings suggest that respondents consider these wide ranges of activities when reporting on how their health affects their UA, and hence the UA dimension measures what it intends to measure. Inquiry into relative importance revealed that housework explains most of the overall variance, followed by leisure, while family activities ranked last.

This sequence of importance ranking was consistent across the majority of disease groups as well as the healthy group, with housework at the top. This finding suggests that problems with housework are more salient and possibly more difficult to adapt too. That is, change in some activities might be less obvious to the individual (e.g., leisure activities change from playing squash to playing chess), but it is difficult to adapt to an inability to do basic housework activities like preparing food or cleaning the house. Another reason for the dominant role of housework might be that the term “usual” is commonly understood as “daily” by respondents [25], which could make other non-daily regular activities (e.g., problems playing golf once a week or meeting family once a month) less likely to be reported when considering one’s usual activities.

Compared to family activities, housework activities contributed twice as much of the overall variation. The possible explanation for the lower contribution of family activities could be an overlap with housework and leisure activities. In the description of the EQ-5D UA dimension, there is no clear definition as what a ‘family activity’ means. Thus, respondents may attribute some of the activities related to family as housework or leisure activities. In fact, it is difficult to draw a line of demarcation between family activities and housework or leisure activities. For instance, cooking can be both housework and family activities as well as leisure activities. Moreover, people may understand family activities as social phenomena instead of usual activities that are more physical, such as housework, which would be in line with the original EQ-6D that described family and leisure activities under the social relationship dimension [4].

The leisure variable is the second-most important for UA dimension that even exceeds the contribution of work/study. The reason why the relative importance of leisure activities generally exceeds that of work/study is most probably that leisure activities extend from the very informal and casual to highly organized and long-lasting activities, while work/study are specific and well defined for each individual.

In the healthy and disease groups, the relative importance ranking of the main predictors of UA dimension was consistent except in the arthritis group. In the arthritis group, leisure activity was most important, followed by work/study, housework, and family. This result can be explained by the nature of the disease and the way the leisure variable was described. That is, the leisure activities variable focuses on the ability to do vigorous activities such as participating in strenuous sports or lifting heavy objects, as well as moving a table or pushing a vacuum cleaner, which are activities that might be especially challenging to individuals with arthritis. In contrast, in the depression group, leisure, and work/study activities switched importance rankings. This result is likely because of the nature of the leisure variable; leisure is tapping health that is more physical. However, the items used to measure work/study include items that explicitly emphasize emotional problems (i.e., feeling depressed or anxious), which could increase the contribution of work/study in the depression group. For instance, a separate regression analysis (not reported here) with work/study activities as two separate variables (emotional health and physical health) revealed that only the latter significantly affect the UA dimension, suggesting that respondents mainly focus on their physical health when reporting problems on the UA. Furthermore, although depression affects motivation to do usual activities, both housework and leisure (as described in this study) are mostly measuring a physical concept. Lastly, for the healthy sample, housework and leisure activities continued to contribute the lion shares to the UA dimension, and work/study activities interestingly ranked last. However, this is not surprising, considering healthy people are usually less concerned about their work/study compared to people with chronic conditions.

It seems evident that the term usual is understood as activities that are performed with some regularity [25]. Thus, one area of ambiguity is the notion of how ‘regular’ is ‘usual’; i.e., the ambiguity in the interpretation of the term ‘usual’ lies in whether ‘usual’ mean ‘daily’ or ‘regular’ (e.g., visiting families every week-end). Another area of ambiguity is whether ‘usual’ covers ‘role’ or other activities, and whether the individual is fulfilled or not, particularly in reference to activities preceeding illness [25]. Furthermore, Rand-Henriksen et al. compared hypothetical and experience based EQ-5D valuations and argued that usual activities could be the least tangible dimension for the general public [26]. The issue of valuation of EQ-5D health states is beyond the scope of the current study; however, the ambiguity in the interpretation of the term ‘usual’ in the UA dimension could complicate the valuation exercise and warrant future studies.

This study has a number of limitations. One limitation is the measure of leisure variable from the SF-36 questionnaire. As discussed, these items measure mainly vigorous activities (e.g., participating in strenuous sports), and has some overlap with housework. Nevertheless, our findings are consistent with a more precise measure of leisure (obtained from the QLQ-C30 instrument). Another limitation could be the lack of a clear distinction between the described activities, such as housework, leisure, and family activities, which highlights the challenge using such a multi-barreled question to measure usual activities. Further, not all variation in UA dimension is accounted for by the explanatory variables used in this study. This indicates that additional activities other than those described here are important for variation in UA. However, the four major activities alone explained over 44 and 59% of the variation in LRM and OLS, respectively, which is quite large in this kind of social study. Lastly, respondents volunteered to participate in the MIC study, something which might lead to self-selection bias. Despite these limitations, this is the first study that has empirically attempted to test whether these activities, in fact, reflect the UA dimension of EQ-5D. Future studies should apply better measures of work/study, housework, family and leisure activities that can minimize conceptual overlap between these variables. Further studies would also be required to identify other important predictor(s) of the UA dimension, which can give insights into what respondents are considering when they report problems on this dimension.

In conclusion, the UA dimension fairly picks up the described activities, such as work/study, housework, family, and leisure activities. In explaining the variation in the UA dimension, housework is relatively most important, followed by leisure, while family is the least important. In a nutshell, the UA dimension of EQ-5D captures what it intends to measure.

## Electronic supplementary material

Below is the link to the electronic supplementary material.

Supplementary file1 (DOCX 43 kb)
