# Supplementary material for: Does the EQ-5D usual activities dimension measure what it intends to measure? The relative importance of work, study, housework, family or leisure activities
Source: Qual Life Res. 2020 Apr 23;29(9):2553–62. doi: 10.1007/s11136-020-02501-w (PMC7434786; doi:10.1007/s11136-020-02501-w)
Supplement: Supplementary file 1 — Supplementary file1 (DOCX 43 kb) [file 11136_2020_2501_MOESM1_ESM.docx]

**Appendix Table A1**. Regression results for UA dimension of EQ-5D with leisure variable from cancer sample

|  | Model 1 | | | | Model 2 | | | |
| --- | --- | --- | --- | --- | --- | --- | --- | --- |
|  |  |  | 95% CI | |  |  | 95% CI | |
| Variables | β | S.E. | Lower | Upper | β | S.E. | Lower | Upper |
| Work/Study | 0.100*** | 0.019 | 0.064 | 0.137 | 0.037*** | 0.006 | 0.026 | 0.048 |
| Housework |  |  |  |  |  |  |  |  |
| Can do relatively easy | 1.511*** | 0.277 | 0.968 | 2.053 | 0.181*** | 0.053 | 0.077 | 0.284 |
| Can do very slowly | 3.798*** | 0.454 | 2.909 | 4.687 | 0.693*** | 0.083 | 0.530 | 0.857 |
| Cannot do most/none | 2.114*** | 0.541 | 1.054 | 3.174 | 0.968*** | 0.135 | 0.703 | 1.233 |
| Family |  |  |  |  |  |  |  |  |
| Some parts affected | 0.573** | 0.251 | 0.081 | 1.064 | 0.005 | 0.061 | -0.114 | 0.125 |
| Many/cannot cary any | 2.654** | 1.101 | 0.497 | 4.812 | 0.416*** | 0.111 | 0.197 | 0.634 |
| Leisure |  |  |  |  |  |  |  |  |
| Limited a little | 1.202*** | 0.273 | 0.668 | 1.737 | 0.229*** | 0.057 | 0.118 | 0.340 |
| Limited quite a bit | 1.266*** | 0.364 | 0.553 | 1.978 | 0.362*** | 0.088 | 0.189 | 0.535 |
| Limited very much | 1.805*** | 0.601 | 0.628 | 2.982 | 0.663*** | 0.116 | 0.434 | 0.891 |
| Age (in years) | 0.001 | 0.011 | -0.020 | 0.022 | -0.001 | 0.002 | -0.004 | 0.003 |
| Gender |  |  |  |  |  |  |  |  |
| Female | -0.125 | 0.256 | -0.628 | 0.377 | -0.037 | 0.043 | -0.122 | 0.048 |
| Marital |  |  |  |  |  |  |  |  |
| Live with spouse/partner | -0.174 | 0.243 | -0.650 | 0.301 | -0.026 | 0.043 | -0.110 | 0.059 |
| Employment status |  |  |  |  |  |  |  |  |
| Unemployed | -0.08 | 0.285 | -0.811 | 0.308 | -0.079 | 0.050 | -0.094 | 0.102 |
| Education |  |  |  |  |  |  |  |  |
| Diploma or certificate | -0.251 | 0.280 | -0.610 | 0.486 | 0.004 | 0.050 | -0.094 | 0.101 |
| University degree | -0.062 | 0.590 | -1.237 | 1.077 | 0.003 | 0.090 | -0.256 | 0.098 |
| Country |  |  |  |  |  |  |  |  |
| Australia | -0.05 | 0.377 | -0.790 | 0.690 | -0.074 | 0.065 | -0.203 | 0.054 |
| Canada | -0.262 | 0.418 | -1.083 | 0.558 | -0.062 | 0.070 | -0.200 | 0.076 |
| Germany | -0.844** | 0.389 | -1.607 | -0.080 | -0.152** | 0.073 | -0.295 | -0.009 |
| Norway | -0.513 | 0.411 | -1.317 | 0.292 | -0.195** | 0.078 | -0.347 | -0.043 |
| USA | -0.934** | 0.395 | -1.708 | -0.161 | -0.140* | 0.073 | -0.283 | 0.004 |
| Constant | -3.085*** | 1.072 | -5.186 | -0.985 | 0.910*** | 0.194 | 0.528 | 1.292 |

*Note*: Model 1 applied Logistic regression, and Model 2 linear regression . *β* estimated coefficients, *S.E*. standard error, *CI* confidence interval
*** p<0.01, ** p<0.05, * p<0.1

**Appendix Table A2**. Regression results for UA dimension of EQ-5D with work/study variable from cancer sample

|  | Model 1 | | | | | Model 2 | | | | |
| --- | --- | --- | --- | --- | --- | --- | --- | --- | --- | --- |
|  |  | |  | 95% Conf. Interval | |  | |  | 95% Conf. Interval | |
| Variables | β | S.E. | | Lower | Upper | β | S.E. | | Lower | Upper |
| Work/study |  |  | |  |  |  |  | |  |  |
| Limited a little | 1.168*** | 0.277 | | 0.624 | 1.712 | 0.268*** | 0.059 | | 0.152 | 0.383 |
| Limited quite a bit | 1.784*** | 0.401 | | 0.998 | 2.571 | 0.527*** | 0.092 | | 0.346 | 0.708 |
| Limited very much | 2.907*** | 0.695 | | 1.544 | 4.270 | 0.971*** | 0.123 | | 0.729 | 1.214 |
| Housework |  |  | |  |  |  |  | |  |  |
| Can do relatively easy | 1.130*** | 0.294 | | 0.553 | 1.706 | 0.150*** | 0.056 | | 0.040 | 0.260 |
| Can do very slowly | 3.217*** | 0.447 | | 2.342 | 4.093 | 0.630*** | 0.095 | | 0.445 | 0.816 |
| Cannot do most/none | 1.305** | 0.609 | | 0.111 | 2.499 | 0.836*** | 0.152 | | 0.538 | 1.134 |
| Family |  |  | |  |  |  |  | |  |  |
| Some parts affected | 0.762*** | 0.251 | | 0.271 | 1.253 | 0.098* | 0.061 | | -0.021 | 0.217 |
| Many/cannot cary any | 3.021*** | 1.019 | | 1.024 | 5.018 | 0.587*** | 0.111 | | 0.369 | 0.805 |
| Leisure |  |  | |  |  |  |  | |  |  |
| Slightly limited | 1.543** | 0.686 | | 0.198 | 2.888 | 0.045 | 0.040 | | -0.033 | 0.123 |
| Somewhat limited | 2.413*** | 0.700 | | 1.041 | 3.785 | 0.224*** | 0.064 | | 0.098 | 0.350 |
| Moderately limited | 2.602*** | 0.708 | | 1.214 | 3.990 | 0.227*** | 0.073 | | 0.084 | 0.370 |
| Limited a lot | 3.135*** | 0.797 | | 1.573 | 4.697 | 0.499*** | 0.116 | | 0.270 | 0.727 |
| Age (in years) | -0.012 | 0.011 | | -0.034 | 0.011 | -0.003 | 0.002 | | -0.007 | 0.001 |
| Gender |  |  | |  |  |  |  | |  |  |
| Female | -0.240 | 0.254 | | -0.739 | 0.258 | -0.050 | 0.043 | | -0.135 | 0.034 |
| Marital |  |  | |  |  |  |  | |  |  |
| Live with spouse/partner | -0.132 | 0.244 | | -0.611 | 0.346 | -0.043 | 0.043 | | -0.128 | 0.042 |
| Education |  |  | |  |  |  |  | |  |  |
| Diploma/certificate | -0.463 | 0.296 | | -1.042 | 0.117 | -0.033 | 0.052 | | -0.135 | 0.069 |
| University | -0.215 | 0.281 | | -0.764 | 0.335 | -0.032 | 0.049 | | -0.129 | 0.065 |
| Employment status |  |  | |  |  |  |  | |  |  |
| Unemployed | 0.047 | 0.585 | | -1.100 | 1.193 | -0.039 | 0.095 | | -0.226 | 0.148 |
| Country |  |  | |  |  |  |  | |  |  |
| Australia | -0.208 | 0.383 | | -0.959 | 0.542 | -0.089 | 0.066 | | -0.219 | 0.042 |
| Canada | -0.424 | 0.408 | | -1.224 | 0.377 | -0.092 | 0.069 | | -0.228 | 0.045 |
| Germany | -0.746* | 0.412 | | -1.554 | 0.061 | -0.159** | 0.076 | | -0.309 | -0.010 |
| Norway | -0.418 | 0.421 | | -1.243 | 0.407 | -0.181** | 0.079 | | -0.336 | -0.027 |
| USA | -0.767* | 0.399 | | -1.549 | 0.015 | -0.121* | 0.075 | | -0.268 | 0.027 |
| Constant | -12.497*** | 3.530 | | -19.415 | -5.579 | 0.399 | 0.285 | | -0.160 | 0.958 |

*Note*: Model 1 applied Logistic regression , and Model 2 linear regression . *β* estimated coefficients, *S.E.* standard error, *CI* confidence interval

*** p<0.01, ** p<0.05, * p<0.1.

**Appendix Table A3.** Relative importance of predictors for EQ-5D UA dimension: Sensitivity analysis

|  | Scenario 1 | | | | Scenario 2 | | | |
| --- | --- | --- | --- | --- | --- | --- | --- | --- |
|  | Model 1 | | Model 2 | | Model 1 | | Model 2 | |
| Predictors | Est. | Per cent | Est. | Per cent | Est. | Per cent | Est. | Per cent |
| Work/study | 0.121 | 23.44 | 0.188 | 27.91 | 0.128 | 24.08 | 0.184 | 27.85 |
| Housework | 0.194 | 37.75 | 0.209 | 31.11 | 0.171 | 31.98 | 0.197 | 29.86 |
| Family | 0.082 | 15.94 | 0.113 | 16.78 | 0.087 | 16.29 | 0.12 | 18.28 |
| Lesiure | 0.103 | 19.93 | 0.147 | 21.91 | 0.134 | 25.13 | 0.143 | 21.76 |
| SDC | 0.004 | 0.8 | 0.006 | 0.85 | 0.005 | 0.88 | 0.005 | 0.8 |
| Country | 0.011 | 2.14 | 0.01 | 1.44 | 0.008 | 1.59 | 0.009 | 1.39 |
| Total | 0.515 | 100 | 0.672 | 100 | 0.533 | 100 | 0.659 | 100 |

*Note*: Scenario 1 and Scenario 2 used Leisure and Work/study variables from cancer samples to test the consistency of results, respectively. Model 1 applied logistic regression, and Model 2 linear regression. *SDC*Socio-demographic characteristics (age, gender, marital, education & unemployment), *Est*. Estimated Shapley value

|  | Healthy group | | Arthritis | | Asthma | | Cancer | | Depression | | Diabetes | | Hearing problem | | Heart problems | |
| --- | --- | --- | --- | --- | --- | --- | --- | --- | --- | --- | --- | --- | --- | --- | --- | --- |
| Predictors | Est. | % | Est. | % | Est. | % | Est. | % | Est. | % | Est. | % | Est. | % | Est. | % |
| Work/Study | 0.044 | 10.72 | 0.132 | 29.43 | 0.124 | 25.43 | 0.119 | 22.64 | 0.095 | 25.22 | 0.116 | 23.22 | 0.091 | 23.74 | 0.120 | 24.65 |
| Housework | 0.130 | 32.10 | 0.089 | 19.74 | 0.147 | 30.03 | 0.176 | 33.51 | 0.100 | 26.53 | 0.156 | 31.15 | 0.102 | 26.46 | 0.157 | 32.38 |
| Family | 0.081 | 19. 96 | 0.057 | 12.75 | 0.061 | 12.54 | 0.082 | 15.50 | 0.056 | 15.03 | 0.066 | 13.21 | 0.070 | 18.25 | 0.064 | 13.26 |
| Leisure | 0.127 | 31.18 | 0.148 | 33.05 | 0.126 | 25.81 | 0.135 | 25.68 | 0.077 | 20.47 | 0.142 | 28.33 | 0.096 | 24.82 | 0.130 | 26.67 |
| SDC | 0.011 | 2.59 | 0.012 | 2.58 | 0.024 | 4.83 | 0.006 | 1.08 | 0.021 | 5.61 | 0.011 | 2.16 | 0.011 | 2.90 | 0.007 | 1.44 |
| Country | 0.014 | 3.45 | 0.011 | 2.44 | 0.007 | 1.35 | 0.008 | 1.60 | 0.027 | 7.15 | 0.010 | 1.94 | 0.015 | 3.83 | 0.008 | 1.60 |
| Total | 0.405 | 100 | 0.449 | 100 | 0.487 | 100 | 0.526 | 100 | 0.375 | 100 | 0.500 | 100 | 0.486 | 100 | 0.486 | 100 |

**Appendix Table A4.** Shapely values decomposition in the healthy and disease groups

Note. *Est*. Estimated Shapley value, *SDC* Socio-demographic characteristics (age, gender, marital, education and unemployment)
